# Porcine parvovirus infection induces necroptosis of porcine placental trophoblast cells via a ZBP1-mediated pathway

**DOI:** 10.1186/s13567-024-01410-x

**Published:** 2024-11-29

**Authors:** Ning Xu, Qian Du, Yijiao Cheng, Lichen Nie, Peipei Ma, Dingwen Feng, Yong Huang, Dewen Tong

**Affiliations:** 1 College of Veterinary Medicine, Northwest A&F University, Yangling, China; 2 Engineering Research Center of Efficient New Vaccines for Animals, Ministry of Education, Yangling, China; 3 Key Laboratory of Ruminant Disease Prevention and Control (West), Ministry of Agriculture and Rural Affairs, Yangling, China; 4 Engineering Research Center of Efficient New Vaccines for Animals, Universities of Shaanxi Province, Yangling, China

**Keywords:** Porcine parvovirus, porcine placental trophoblast cells, necroptosis, Z-nucleic acid-binding protein 1

## Abstract

Porcine parvovirus (PPV) infection induces germ cell death, leading to reproductive disorders in first-pregnant sows. Porcine placental trophoblast cells (PTCs) are the major target of PPV, and we have previously found that PPV infection leads to the death of PTCs by a non-apoptotic process, which may be related to PPV pathogenicity. The Z-nucleic acid-binding protein 1 (ZBP1) is often activated after virus invasion and mediates subsequent cell death. Here, we found that PPV infection induced ZBP1-mediated necroptosis of porcine PTCs in the presence of the apoptosis inhibitor, AC-DEVD-CHO. ZBP1 expression was upregulated during PPV infection, and ZBP1 knockout or RNA interference significantly reduced its expression along with the PPV-induced necroptosis. We discovered that RIPK3 and MLKL, but not Caspase-8, were involved in downstream signaling of ZBP1 during PPV infection; the phosphorylation levels of RIPK3 and MLKL were enhanced, but Caspase-8 was not significantly cleaved. The knockout of RIPK3 and MLKL significantly reduced the PPV infection-induced necroptosis of porcine PTCs. RIPK3 knockout did not affect the PPV infection-induced upregulation of ZBP1 expression, but blocked the activation of MLKL. MLKL knockout did not affect the upregulation of ZBP1 expression and RIPK3 phosphorylation during PPV infection. UV-inactivated PPV induced significantly less necroptosis of porcine PTCs than non-irradiated PPV. A PPV strain with a mutation in the translation initiation codon was still able to induce necroptosis of PTCs through the ZBP1/RIPK3/MLKL pathway. These results provide new insights into the pathogenic mechanism of PPV infection and suggest that PPV infection of porcine PTCs induces necroptosis through the viral DNA-dependent ZBP1/RIPK3/MLKL pathway.

**Supplementary Information:**

The online version contains supplementary material available at 10.1186/s13567-024-01410-x.

## Introduction

Viruses usually induce cell death to facilitate their spreading from one host cell to another, and the virus-induced cell death causes lesions in infected organs. Porcine parvovirus (PPV) is a common virus with a small single-stranded DNA genome that induces reproductive disorders, especially in first-pregnant sows. In previous studies, we found that PPV infected porcine placental trophoblast cells (PTCs), inducing apoptosis and autophagy [1, 2]. However, it has also been observed that PPV infection can lead to regionalized mineralized lesions of the pig placenta that are not the result of apoptosis or autophagy [3]. In a previous study, we found that PPV infection could also lead to the death of PTCs in a non-apoptotic way [1], but the mechanism was not elucidated. The goal of this paper was to characterize this type of PPV infection-induced cell death and the cellular pathways involved.

The interferon-induced protein, Z-nucleic acid-binding protein 1 (ZBP1), also known as DNA-dependent activator of IFN regulatory factors (DAI and DLM-1), is a sensor of the “Z-form” of nucleic acid (Z-DNA and Z-RNA) in cells. Studies have found that ZBP1 acts as an innate sensor for virus infection and triggers multiple programmed cell death pathways including apoptosis, necroptosis, and pyroptosis [4, 5]. Upon viral invasion, ZBP1 senses viral nucleic acids through its two N-terminal Z-nucleic acid-binding domains (Zα1 and Zα2) and recruits receptor-interacting protein kinase 3 (RIPK3) via an RIP homotypic interaction motif (RHIM)-mediated binding [6]. The ZBP1-RIPK3 complex then induces necroptosis mediated by mixed lineage kinase domain-like (MLKL), apoptosis caused by FAS-associated death domain (FADD)-Caspase-8, or pyroptosis mediated by the NOD-, LRR- and pyrin domain-containing protein 3 (NLRP3) inflammasome [7]. Recently, it has been found that influenza A virus infection can induce apoptosis, necroptosis and pyroptosis through ZBP1-mediated NLRP3 activation of inflammasomes in cells [8]. Herpes simplex virus 1 also induces ZBP1-mediated apoptosis, necroptosis and pyroptosis of cells during infection [9]. Hepatitis B virus (HBV) relies on ZBP1-mediated necroptosis during the progression from chronic HBV infection (CHI) to HBV-related hepatic fibrosis (HBV-HF) and HBV-related hepatocellular carcinoma (HBV-HCC) [10]. Thus, we hypothesized that ZBP1 plays a role in the non-apoptotic cell death induced in porcine PTCs by PPV infection.

In the present study, we found that the PPV infection-induced non-apoptotic death of porcine PTCs possessed the characteristics of necroptosis, involving the activation of MLKL and the release of LDH. We showed that the expression of ZBP1 was significantly upregulated in PPV-infected PTCs, and that ZBP1 deficiency attenuated PPV-induced necroptosis. PTCs with separate knockouts of RIPK3, MLKL, and Caspase-8 were generated, and we found that PPV infection induced necroptosis of PTCs through the ZBP1/RIPK3/MLKL pathway. Ultimately, we discovered that, during the PPV infection process in PTCs, PPV DNA binds to ZBP1, which initiates the ZBP1/RIPK3/MLKL signaling cascade and induces necroptosis. Thus, our results revealed the specific mechanism underlying the triggering of necroptosis in PTCs by PPV infection.

## Materials and methods

### Cells and virus

The porcine placental trophoblast cells (PTCs) were isolated from healthy gilts and immortalized by telomerase reconstitution as described in our previous study [11]. The PTCs were cultured in medium 199 (11150059, Gibco, USA) supplemented with 10% FBS (FSP500, ExCell Bio, China), 100 U/mL penicillin and 100 µg/mL streptomycin (P1400, Solarbio, China), at 37 °C in a 5% CO_2_ incubator. The PPV XY strain (Genbank: MK993540) was propagated in porcine kidney-15 (PK15) cells, and the titer was 10^6.12^ TCID_50_/0.1 mL. Fibrinogen (F3879, Sigma-Aldrich, USA) was dissolved in a 0.9% sodium chloride solution to achieve a final concentration of 10 mg/mL, the fibrinogen solution was then mixed with PPV in a volume ratio of 9:1 and loaded into a transparent bag for UV irradiation, when the irradiation dose reached 900 J/m^2^, the titer of PPV virus decreased by 4 log s, resulting in the generation of UV-inactivated PPV. Viral titers were determined as 50% tissue culture infectious dose (TCID_50_) measurement in PTCs according to the Reed-Muench method.

### Inhibitors and antibodies

The apoptosis inhibitor, Ac-DEVD-CHO (C1206), was purchased from Beyotime Biotech (Shanghai, China). Mouse anti-VP2 monoclonal antibody (bsm-41390M) was purchased from Bioss (Beijing, China). Mouse anti-ZBP1 monoclonal antibody (sc-271483) and mouse anti-RIPK3 monoclonal antibody (sc-374639) were purchased from Santa Cruz (USA). Rabbit anti-p-RIPK3 monoclonal antibody (93654S) was purchased from CST (Danvers, MA, USA). Mouse anti-Caspase-8 monoclonal antibody (ab32397) was purchased from Abcam (England). Rabbit anti-MLKL polyclonal antibody (21066-1-AP) was purchased from Proteintech (Wuhan, China). Rabbit anti-p-MLKL monoclonal antibody (AP1244) was purchased from ABclonal (Wuhan, China). Mouse anti-β-actin monoclonal antibody (13C000500) was purchased from GenScript (Nanjing, China).

### Transmission electron microscopy

Porcine PTCs were mock-infected or infected with PPV at a multiplicity of infection (MOI) of 1 for 72 h, after which the cells were washed with PBS and fixed in 0.1 M sodium phosphate buffer (pH 7.4) containing 2.5% glutaraldehyde and 4% paraformaldehyde for 5 h at 4 ℃. The cells were harvested and washed with PBS, followed by post-fixation in 2% osmium tetroxide and dehydration by serial washing in 50%, 70%, 90%, 95%, and 100% ethanol. The samples were embedded, cut into thin sections, and viewed with a transmission electron microscope (FEI Inc.).

### Lactate dehydrogenase cytotoxicity assay

The cells were mock-infected or infected with PPV at a MOI of 1 for the indicated time in the presence of the apoptosis inhibitor, Ac-DEVD-CHO, and then the release of lactate dehydrogenase (LDH) was measured using an LDH cytotoxicity assay kit (C0016, Beyotime Biotech) according to the instructions.

### Western blot

The cells were lysed with RIPA buffer containing 1 mM phenylmethylsulfonyl fluoride (PMSF) and a protease inhibitor cocktail. Equal amounts of protein from each extract were separated by SDS-PAGE and then transferred to a polyvinylidene difluoride (PVDF) membrane. After blocking with PBS containing 5% skim milk and 0.1% Tween-20 for 2 h at room temperature, the membrane was incubated with primary antibody overnight at 4 °C, washed, and then incubated with the corresponding HRP-conjugated secondary antibody for 1 h at room temperature. The membrane was incubated with enhanced chemiluminescence (ECL) reagents and exposed in the dark, the primary protein bands were identified, and their intensity was quantitated with Image J software.

### Immunofluorescence assay of MLKL by confocal microscopy

To examine the cellular localization of MLKL, cultured porcine PTCs were mock-infected or infected with PPV at a MOI of 1 for 72 h, then washed with PBS and fixed with ice-cold 4% (wt/vol) paraformaldehyde for 20 min at room temperature, followed by incubation with 0.1% Triton X-100 for 20 min. The cells were incubated with anti-MLKL Abs, followed by the corresponding fluor-conjugated secondary Ab. The nuclei were stained with 4, 6-diamidino-2-phenylindole (DAPI) and fluorescence was observed with a laser scanning confocal microscope (Leica, TCS SP8).

### ZBP1 overexpression

The gene fragment of codon-optimized ZBP1 (GenBank: NM_001123216.1) was synthesized by GeneWiz (Suzhou, China) and cloned to pCAGGS (Invitrogen, CA, USA) for mammalian cell expression. The recombinant vector pCAGGS-ZBP1 was transfected into PTCs using Lipofectamine™ 2000 (Invitrogen) for a duration of 24 h, prior to subsequent PPV infection and Western blot analysis to detect protein associated with the ZBP1 signaling pathway.

### ZBP1 mRNA expression levels by real-time quantitative PCR (RT-qPCR)

Total RNA was extracted from PTCs with TRIzol, verified by 260/280 ratio, and reverse-transcribed into cDNA using a kit. The relative levels were determined by RT-qPCR using the following primers: ZBP1-F: ATGGCAACGAGATGAGACT; ZBP1-R: AGGAAGCACGAGCGAATT.

### Silencing of ZBP1 in PTCs by transfection with siRNA

The ZBP1-specific siRNA was synthesized by Synon Biotechnology Co., Ltd, (China) and the sequences were 5′-CACCAGCAAAGAAACACCUTT-3′ (siZBP1-638), 5′-GCAACUGUUAGAGAAAGCATT-3′ (siZBP1-759), and 5′-GUCUGAATCCACGAUUGGAGT-3′ (siZBP1-1029). The porcine PTCs were seeded in 6-well or 96-well plates at a density of 2 × 10^5^ cells/well or 2 × 10^4^ cells/well, respectively. After adhesion, the cells were transfected with 40 pmol of ZBP1 siRNA per well in a 6-well plates and 4 pmol of ZBP1 siRNA per well in a 96-well plates using Lipofectamine 6000 transfection reagent (C0526, Beyotime Biotechnology, China) according to the manufacturer’s instructions.

### Generation of ZBP1, Caspase-8, RIPK3, and MLKL knockout PTCs

Three pairs of guide RNAs (gRNAs) were designed for each porcine gene: ZBP1, Caspase-8, RIPK3, and MLKL and are shown in Additional file 7. The gene knockout identification primers used are listed in Additional file 8. The synthesized gRNAs were phosphorylated and subcloned into the lentiCRISPRv2 vector (#52961). The recombinant plasmid and the packaging plasmids, psPAX2 and pMD2.G, were co-transfected into HEK293T cells, and the recombinant lentiviruses were collected 72 h after transfection. The PTCs were infected with the recombinant lentiviruses for 48 h, and then selected with puromycin. Positive cells were obtained and transferred to 96-well plates for monoclonal growth.

### Statistical analysis

All experiments were independently performed three times, and the results are representative of two or three independent experiments. Data are expressed as mean ± SD and analyzed by ANOVA or unpaired Student’s *t* tests. Statistical significance was defined as *P* < 0.05 or *P* < 0.01.

## Results

### PPV infection of porcine PTCs induces necroptosis

**Figure 1:**
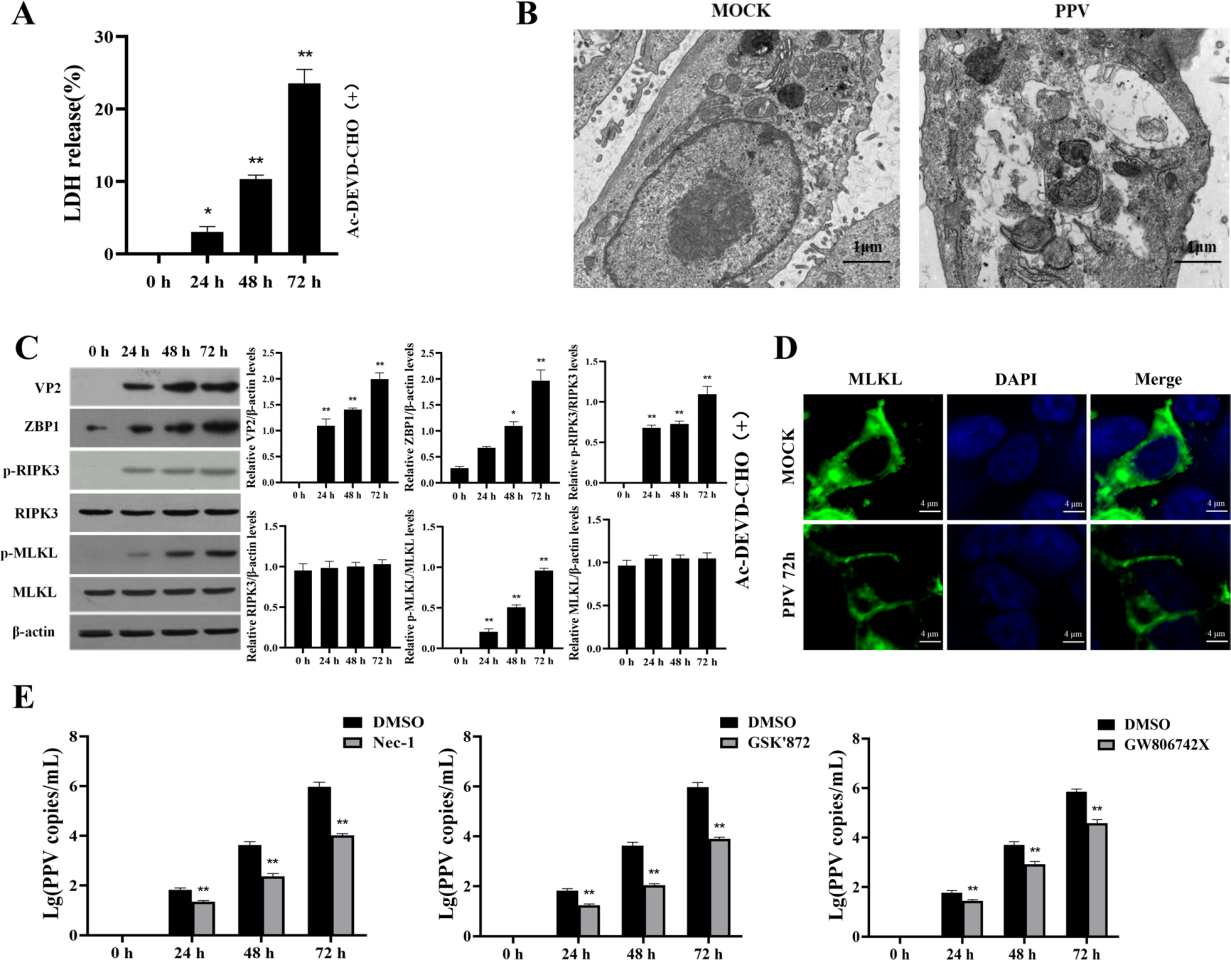
**PPV infection induces both apoptotic and necroptotic cell death in porcine PTCs**. **A**–**D** Porcine PTCs were pretreated with the apoptosis inhibitor, Ac-DEVD-CHO, for 1 h and subsequently infected with PPV at a MOI of 1 for 0, 24, 48, and 72 h in the presence of Ac-DEVD-CHO. **A** Cellular LDH release levels were measured. **B** The MOCK- and PPV-infected cells were observed by transmission electron microscopy at 72 h. **C** The expression levels of PPV VP2, ZBP1, p-RIPK3, RIPK3, p-MLKL, and MLKL were analyzed by Western blotting. **D** The subcellular localization of MLKL in MOCK- and PPV-infected cells was visualized by confocal microscopy. **E** PTCs were pretreated with the necroptosis inhibitors (Nec-1, 10 μM; GSK'872, 20 μM; GW806742X, 20 nM) or same volume of DMSO for 4 h, respectively, and then infected with 1 MOI PPV for 0 h, 24 h, 48 h, and 72 h in the presence of these inhibitors. The copy numbers of PPV were measured by quantitative PCR. *P < 0.05, **P < 0.01 versus PPV-infected cells at 0 h in **A** and **C**. *P < 0.01 versus DMSO-treated cells in **E**.

In a previous study, we showed that PPV infection induced non-apoptotic cell death of porcine PTCs [1]. To identify the type of non-apoptotic cell death observed here, we pretreated porcine PTCs with an apoptosis inhibitor or not and infected the cells with PPV. The infected cells released LDH after 24 h and showed significant increases at 48 h and 72 h post-infection (Figure 1A, Additional file 1). The TEM results showed that PTCs pretreated with apoptosis inhibitors exhibited organelles swelling, vacuolization, and rupture 72 h after PPV infection (Figure 1B). Since the cells killed by PPV infection appeared to exhibit typical necrotic lesions, we assayed for the activation of RIPK3 and MLKL in the PPV-infected PTCs treated or not treated with apoptosis inhibitors, respectively. We found a significant increase in the expression of the PPV structural protein, VP2, in both groups of PTCs with prolonged infection. Simultaneously, there was a significant upregulation in the phosphorylation of RIPK3 and MLKL, with higher levels in cells infected with PPV for 72 h compared to those infected for 24 and 48 h (Figure 1C, Additional file 2). We also found that in PTCs with apoptosis inhibited, MLKL was mainly translocated near the cytomembrane in the PPV-infected PTCs at 72 h post-infection, while MLKL was distributed in the cytoplasm of the mock-infected PTCs (Figure 1D). To further clarify whether necroptosis impacts PPV infection, we pretreated the PTCs with the necroptosis inhibitors (Nec-1, 10 μM; GSK'872, 20 μM; GW806742X, 20 nM) for 4 h, respectively, and then infected the cells with 1 MOI PPV for 0 h, 24 h, 48 h, and 72 h in the presence of the inhibitors. The results showed that the PPV copy numbers in the inhibitor-treated cells were significantly lower than that in the DMSO-treated cells (Figure 1E). These results indicate that PPV infection can induce necroptosis in PTCs to promote viral replication.

### ZBP1 mediates PPV-induced necroptosis in porcine PTCs

**Figure 2:**
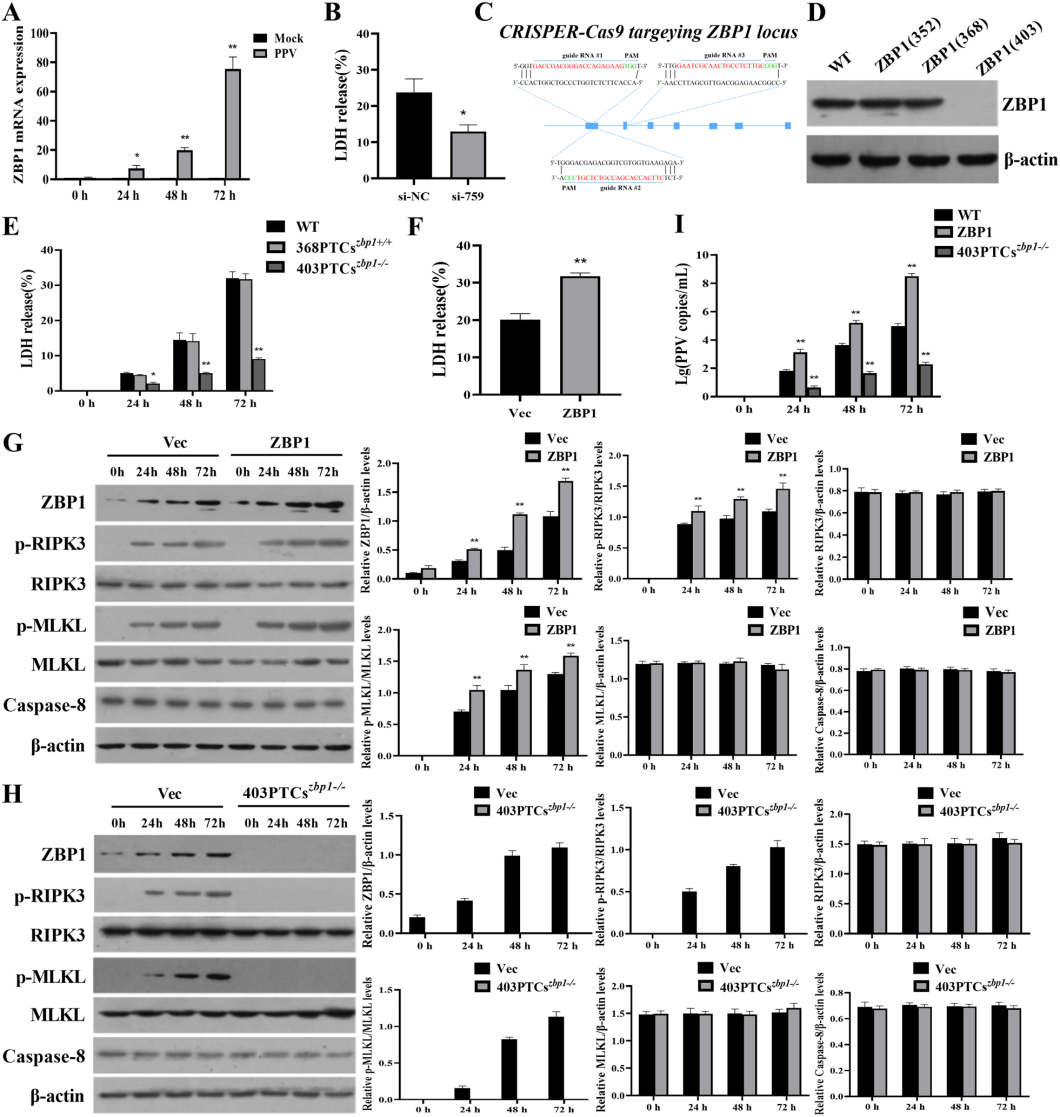
**ZBP1 mediates PPV-induced necroptosis in PTCs in a dose-dependent manner.** **A** Porcine PTCs were pretreated with Ac-DEVD-CHO for 1 h, followed by infection with PPV at a MOI of 1 for 0, 24, 48, and 72 h. ZBP1 mRNA levels were assessed by RT-qPCR. **B** PTCs were transfected with ZBP1-specific siRNA (siZBP1-759) and negative control (siNC) for 48 h, and LDH release was measured after PPV infection. **C** Targeting sites of the three ZBP1-specific guide RNAs. **D** Western blot measurement of ZBP1 protein expression in a ZBP1-knockout strain of PTCs. **E** Pretreatment with the apoptosis inhibitor, Ac-DEVD-CHO, for one hour, followed by infection of WT PTCs, 368PTCs^*zbp1*+*/*+^ and 403PTCs^*zbp1−/−*^ with PPV at a MOI of 1. Levels of released LDH were measured. **F** PTCs were transfected with a plasmid expressing ZBP1 at a dose of 2 µg for 24 h, then infection with PPV at a MOI of 1. Levels of released LDH were measured. **G** The wild-type PTCs and ZBP1 overexpression PTCs were infected with PPV at a MOI of 1 for 0, 24, 48, and 72 h. The expression levels of ZBP1, p-RIPK3, RIPK3, p-MLKL, MLKL, and Caspase-8 were determined by Western blot and calculated. **H** The wild-type PTCs and 403PTCs^*zbp1−/−*^ were infected with PPV at a MOI of 1 for 0, 24, 48, and 72 h. The expression levels of ZBP1, p-RIPK3, RIPK3, p-MLKL, MLKL, and Caspase-8 were determined by Western blot and calculated. **I** The copy numbers of PPV in the ZBP1 overexpression PTCs and 403PTCs^*zbp1−/−*^ were measured by quantitative PCR. *P < 0.05, **P < 0.01 versus PPV-uninfected cells in **A**. *P < 0.05 versus siNC-transfected cells in **B**. *P < 0.05, **P < 0.01 versus WT cells in **E**. **P < 0.01 versus vector control cells in **F** and **G**. **P < 0.01 versus WT cells in (**I**).

Since ZBP1 has been reported to play a significant role in the induction of necroptosis in virus-infected cells, we measured the expression levels of ZBP1 in porcine PTCs during PPV infection and found that ZBP1 mRNA was consistently increased (Figure 2A). To knock down the expression of ZBP1, we designed three ZBP1-specific siRNAs, and observed significant reduction in both ZBP1 mRNA and protein expression after si-ZBP1 (759) treatment of porcine PTCs (Additional files 3A and B). We also determined that si-ZBP1 (759) could reduce the LDH release from PTCs at 72 h post-infection (Figure 2B). To further confirm the role of ZBP1 in the PPV infection-induced necroptosis of porcine PTCs, we designed three guide RNAs, ZBP1-352, ZBP1-368, and ZBP1-403, targeting the different sites of the porcine *ZBP1* gene exons to generate PTC lines with *ZBP1* knockout using the CRISPR/Cas9 system (Figure 2C). The results showed that a PTC line, 403PTCs^*zbp1−/−*^, with ZBP1 knockout mediated by ZBP1-403 was successfully obtained. ZBP1 expression was not significantly affected by ZBP1-352 or ZBP1-368, which was confirmed by Western blot assays and sequencing (Figure 2D). We also found that the ZBP1 knockout did not significantly affect PTC viability (Additional file 4). The LDH results showed that 403PTCs^*zbp1−/−*^ exhibited a sustained decrease in LDH release compared to PPV-infected WT PTCs, while the 368PTCs^*zbp1*+*/*+^ released the same level of LDH as WT PTCs (Figure 2E). To further investigate the role of ZBP1 during PPV infection in PTCs, we constructed a plasmid overexpressing ZBP1 and transfected it into PTCs, followed by PPV infection. The results showed that ZBP1 overexpression significantly increased LDH release level in PTCs (Figure 2F). Meanwhile, we examined the activation of the ZBP1/RIPK3/MLKL signaling pathway in PTCs with ZBP1 overexpression and knockout, respectively. The results showed that the levels of p-RIPK3 and p-MLKL were significantly increased in the ZBP1 overexpression PTCs comparing with the vector control transfected cells (Vec) during PPV infection, while the expression levels of RIPK3, MLKL, and Caspase-8 did not significantly change (Figure 2G). In the ZBP1 knockout PTCs (403PTCs^*zbp1−/−*^), the expression of ZBP1, p-RIPK3, and p-MLKL were undetectable, and the levels of RIPK3, MLKL, and Caspase-8 did not significantly change (Figure 2H). Furthermore, we assessed the effect of ZBP1 overexpression and knockout on PPV infection. The results showed that ZBP1 overexpression obviously promoted PPV replication, while ZBP1 knockout significantly inhibited PPV replication (Figure 2I). These findings confirmed that PPV infection upregulates ZBP1 expression, and alter the expression of ZBP1 significantly influence the PPV infection-induced necroptosis as well as viral replication in porcine PTCs, suggesting ZBP1 serves as a crucial cellular factor in PPV-induced necroptosis in porcine PTCs.

### PPV infection activates RIPK3 and MLKL, but not Caspase-8 in porcine PTCs

**Figure 3:**
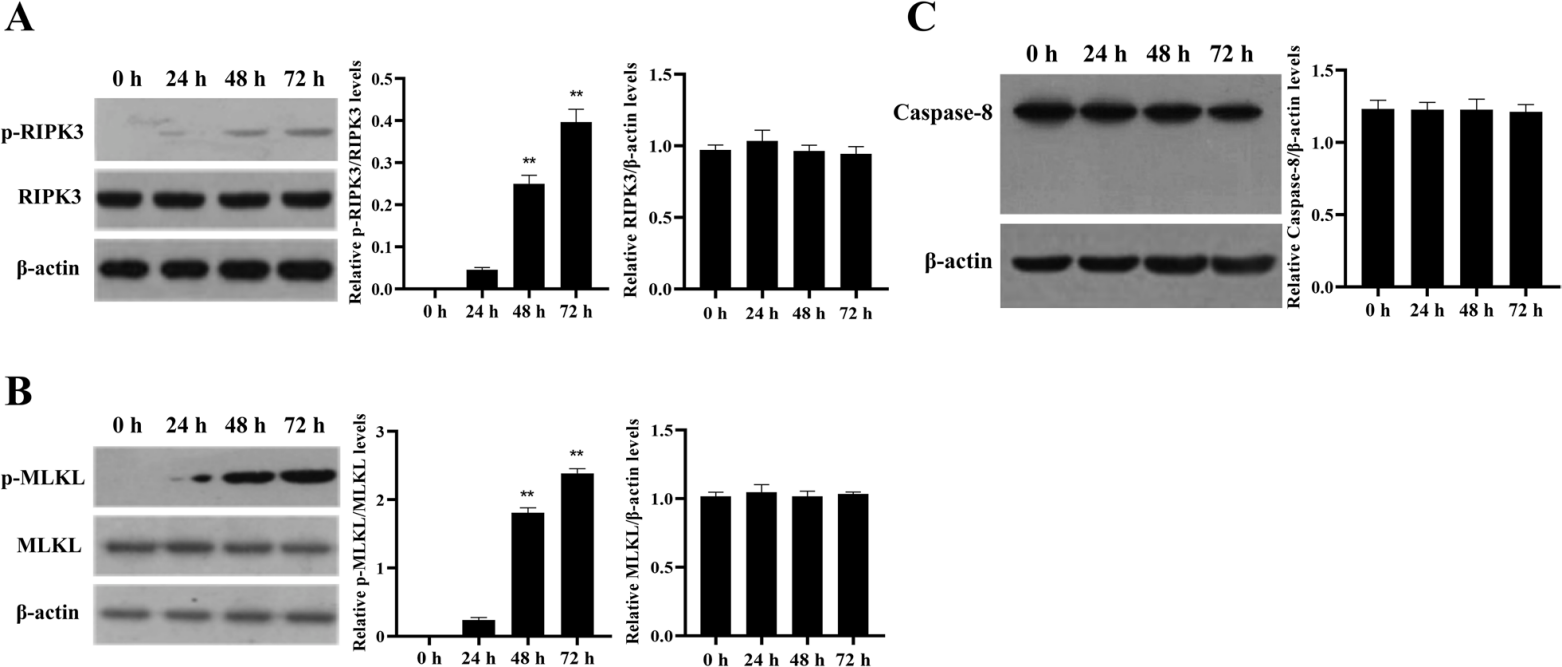
**PPV infection induces non-apoptotic cell death in PTCs associated with RIPK3 and MLKL, but not Caspase-8**. **A**–**C** Pretreatment with the apoptosis inhibitor, Ac-DEVD-CHO, for one hour, followed by infection with PPV at a MOI of 1 for 0, 24, 48, and 72 h in the absence of Ac-DEVD-CHO. Western blotting was performed to assess (**A**) the phosphorylation of RIPK3, (**B**) the phosphorylation of MLKL, and (**C**) the expression and cleavage of Caspase-8. **P < 0.01 versus cells infected by PPV for 0 h in **A** and **B**.

It was reported that ZBP1 mediated necroptosis through RIPK3/MLKL signaling, and that the apoptosis program could switch to necroptosis after cleavage of Caspase-8 [12]. To determine if PPV infection induced ZBP1-mediated necroptosis of porcine PTCs through activation of these proteins, we measured the levels of phosphorylated RIPK3 (p-RIPK3), phosphorylated MLKL (p-MLKL) and the expression and cleavage of Caspase-8. PPV infection induced a significant increase in p-RIPK3 and p-MLKL 24 h post-infection when apoptosis was inhibited (Figures 3A and B); however, the expression and cleavage of Caspase-8 was not changed (Figure 3C). These results indicate that PPV infection induces necroptosis of porcine PTCs through the activation of RIPK3 and MLKL, but not Caspase-8.

### Knockout of RIPK3 does not affect PPV infection-mediated upregulation of ZBP1 expression, but inhibits MLKL activation

**Figure 4:**
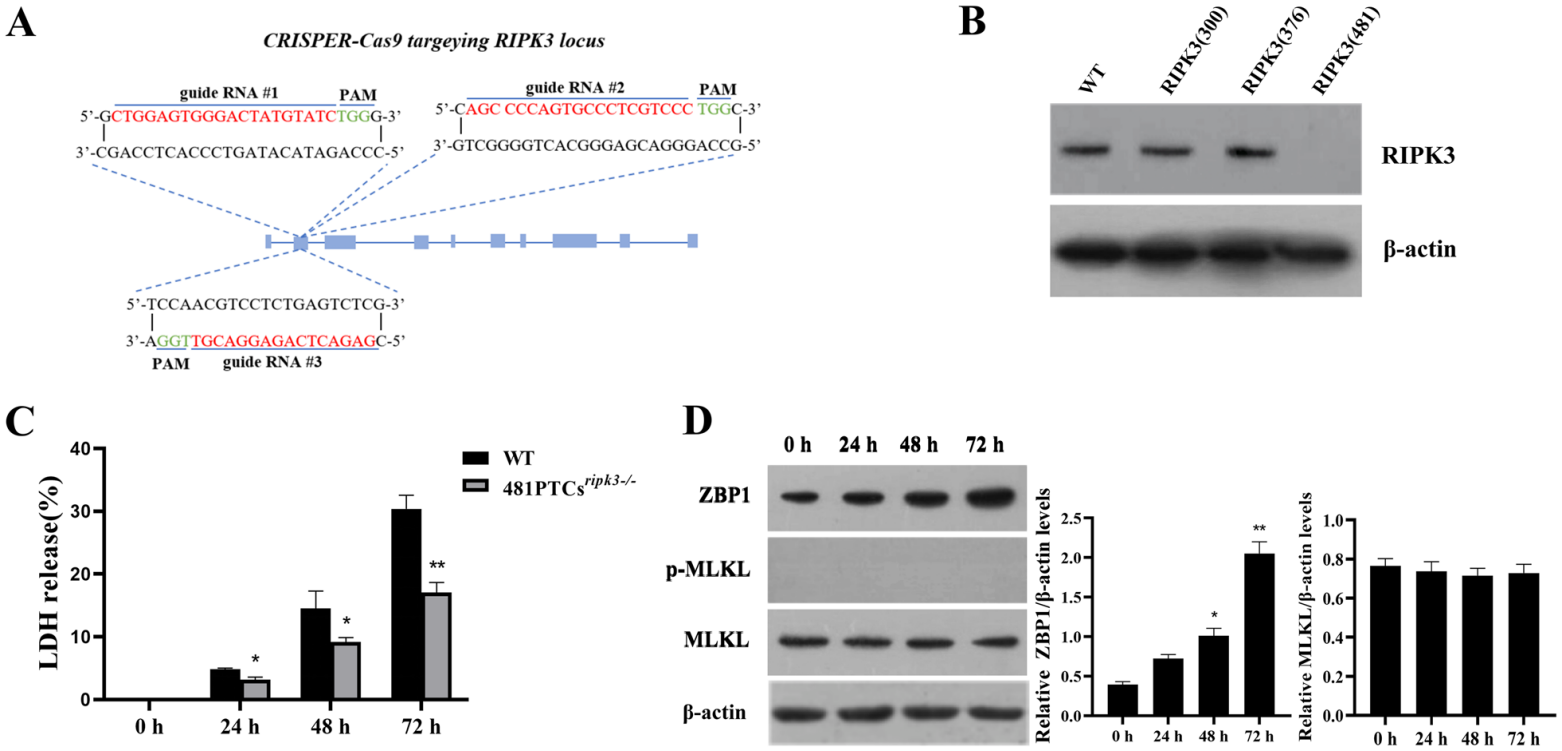
**Inhibiting RIPK3 expression does not affect ZBP1 expression, but blocks MLKL phosphorylation**. **A** The targeting sites of the three RIPK3-specific guide RNAs. **B** Western blot determination of the expression of RIPK3 in PTCs with RIPK3-knockout by CRISPR/Cas9. **C**, **D** 481PTCs^*ripk3−/−*^ cells and WT PTCs were pretreated with the apoptosis inhibitor, Ac-DEVD-CHO, for one hour and then infected with PPV at a MOI of 1 for 0, 24, 48, and 72 h in the absence of Ac-DEVD-CHO. **C** Level of released LDH. **D** Western blot determination of the protein expression levels of ZBP1, p-MLKL, and MLKL. *P < 0.05, **P < 0.01 versus WT cells in **C**. *P < 0.05, **P < 0.01 versus cells infected by PPV for 0 h in **D**.

To further investigate the role of RIPK3 during PPV infection-induced cell necroptosis, we used CRISPR/Cas9 to generate a porcine PTC line with a RIPK3 knockout. Three distinct guide RNAs, RIPK3-300, RIPK3-376, and RIPK3-481, targeting different loci within the RIPK3 gene exons were designed, synthesized (Figure 4A), and introduced into porcine PTCs along with Cas9 using lentivirus vectors. Test results showed that the expression of RIPK3 was successfully inhibited by RIPK3-481, but only partially reduced using RIPK3-300 and RIPK3-376 (Figure 4B). Sequencing confirmed depletion of the RIPK3 gene in the RIPK3-418-treated cells (481PTCs^*ripk3−/−*^) (Additional file 5A). Viability experiments showed that the RIPK3 gene depletion did not significantly affect the growth of 481PTCs^*ripk3−/−*^ (Additional file 5B). The LDH results showed that during PPV infection, 481PTCs^*ripk3−/−*^ exhibited a sustained decrease in LDH release compared to WT PTCs, and the LDH levels were significantly lower at 48 h and 72 h post-infection (Figure 4C). We also examined the impact of RIPK3 gene knockout on the expression of ZBP1 as well as the activation of MLKL. At 24 h after PPV infection, ZBP1 expression was still significantly increased in 481PTCs^*ripk3−/−*^, however, there was no measurable expression of p-MLKL throughout the entire infection process (Figure 4D). Thus, we conclude that knockout of RIPK3 in porcine PTCs does not affect the PPV infection-induced upregulation of ZBP1, but does hinder the activation of MLKL.

### Knockout of MLKL does not affect the upregulation of ZBP1 expression or the activation of RIPK3 in PPV-infected porcine PTCs

**Figure 5:**
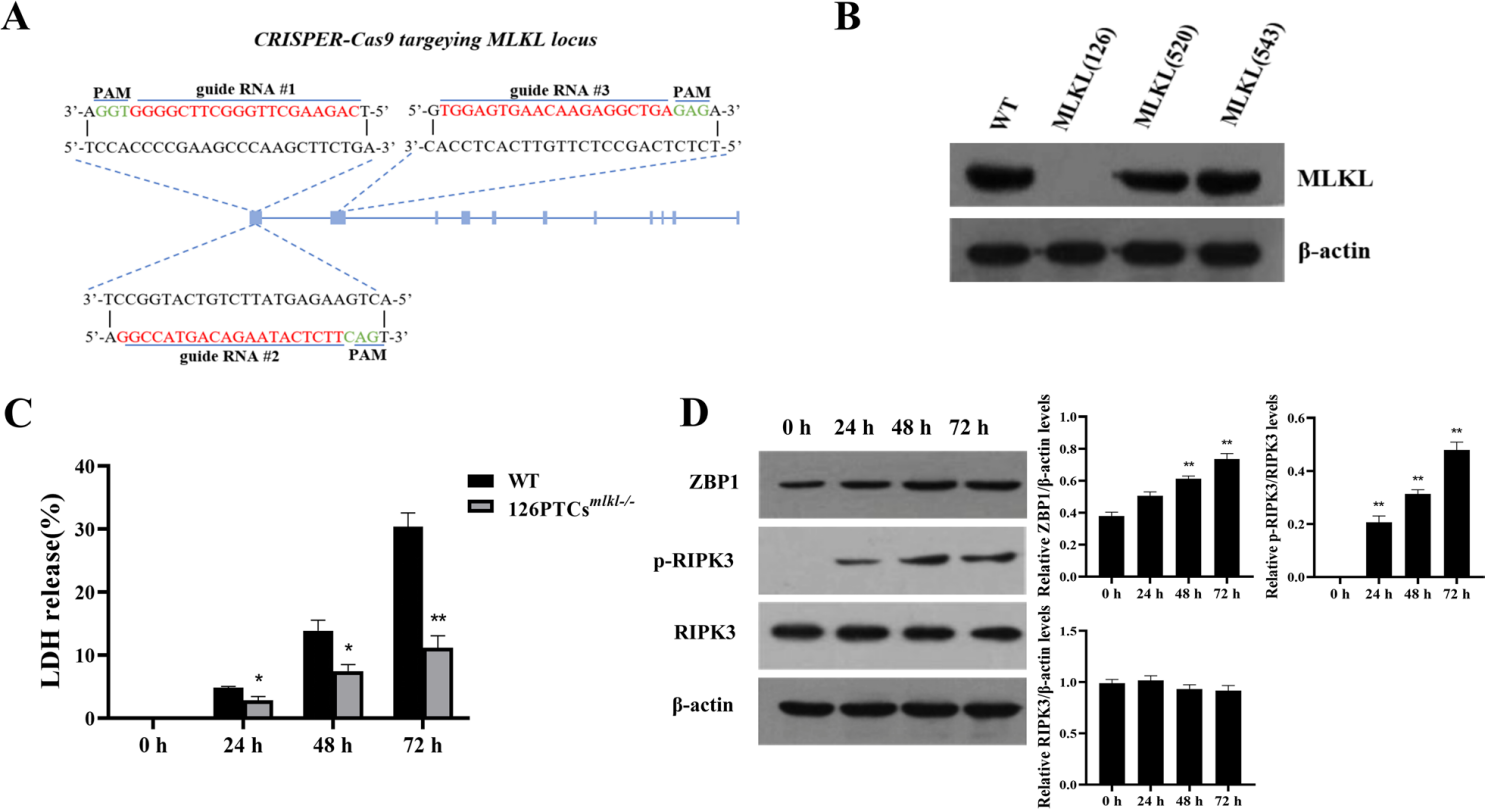
**Inhibiting MLKL expression does not affect upregulation of ZBP1 or phosphorylation of RIPK3.** **A** Targeting sites of the three MLKL-specific guide RNAs. **B** Western blot determination of the expression of MLKL in PTCs with MLKL-knockout by CRISPR/Cas9. **C**, **D** 126PTCs^*mlkl−/−*^ cells and WT PTCs were pretreated with the apoptosis inhibitor, Ac-DEVD-CHO, for one hour and then infected with PPV at a MOI of 1 for 0, 24, 48, and 72 h in the absence of Ac-DEVD-CHO. **C** Level of released LDH. **D** Western blot determination of the protein expression of ZBP1, p-RIPK3, and RIPK3. *P < 0.05, **P < 0.01 versus WT cells in **C**. **P < 0.01 versus cells infected by PPV for 0 h in **D**.

We also generated a porcine PTC line with an MLKL knockout (126PTCs^*mlkl−/−*^) using the CRISPR/Cas9 system with an MLKL-126 guide RNA (Figures 5A, B and Additional file 6A). The MLKL knockout did not significantly affect viability (Additional file 6B), and PPV-infected 126PTCs^*mlkl−/−*^ released significantly less LDH than WT PTCs (Figure 5C). The expression of ZBP1 and the activation of RIPK3 during PPV infection was also unaffected by MLKL knockout in PTCs (Figure 5D). These findings support the hypothesis that MLKL is the effector protein of the ZBP1-mediated necroptosis induced by PPV infection of porcine PTCs.

### UV inactivation of PPV significantly reduces infection-induced necroptosis, while initiation codon mutations do not

**Figure 6:**
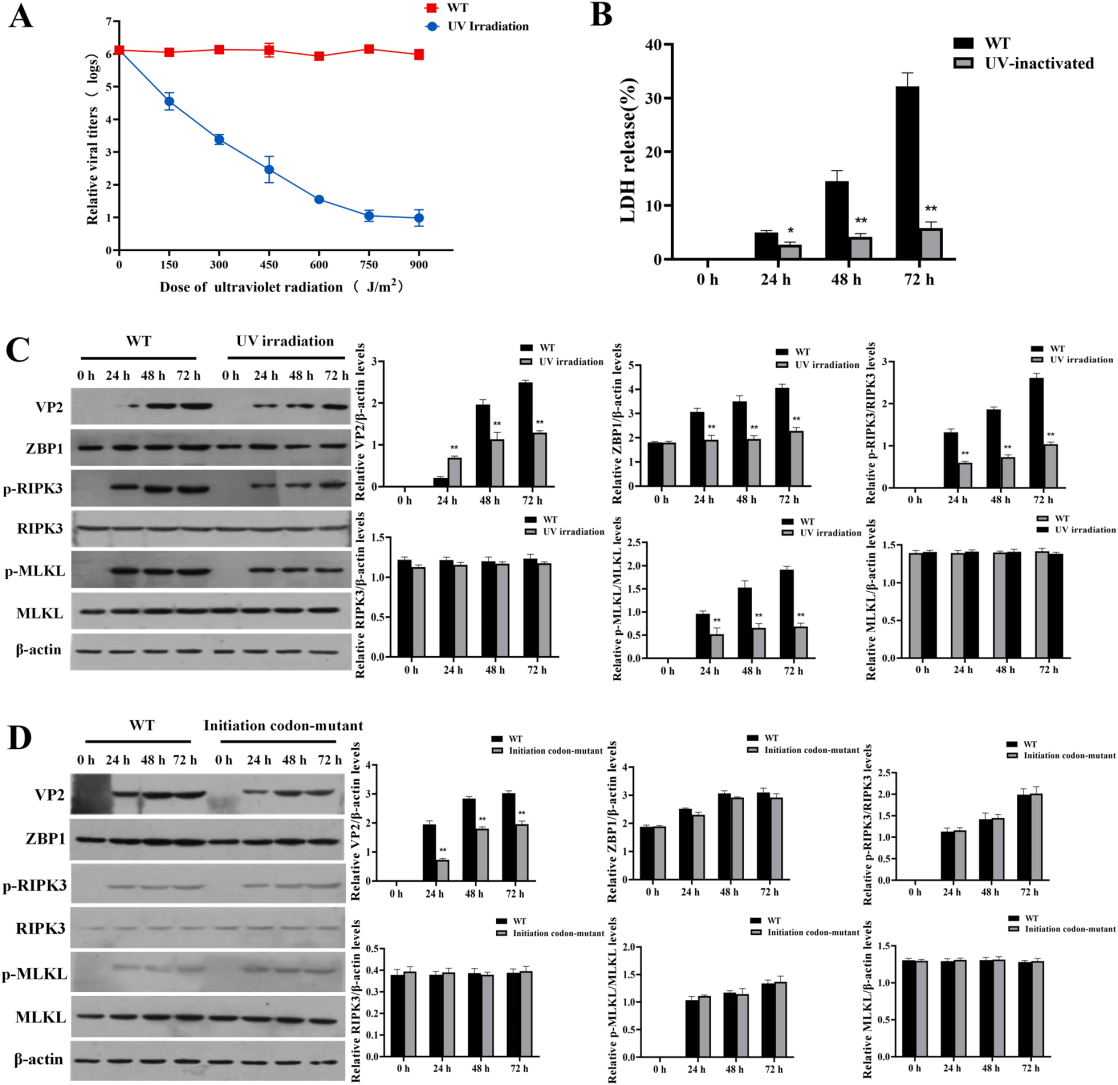
**Disruption of PPV DNA significantly reduces necroptosis. A** Relative viral titers of UV-inactivated PPV were determined using the Reed-Muench method. **B**, **C** Porcine PTCs were pretreated with the apoptosis inhibitor, Ac-DEVD-CHO, for 1 h and subsequently infected with PPV at a MOI of 1 or an equivalent dose of UV-inactivated PPV for 0, 24, 48, and 72 h in the absence of Ac-DEVD-CHO. **B** Levels of released LDH were measured. **C** Western blot determination of protein expression of ZBP1, p-RIPK3, RIPK3, p-MLKL, and MLKL in PTCs infected with UV-inactivated PPV. **D** Western blot determination of protein expression levels of ZBP1, p-RIPK3, RIPK3, p-MLKL, and MLKL in PTCs transfected with initiation codon-mutated PPV. *P < 0.05, **P < 0.01 versus WT cells in **B**. **P < 0.01 versus WT cells in **C** and **D**.

Since ZBP1 is the sensor of Z-DNA and Z-RNA, we investigated whether the PPV genome or the transcriptome was the activator of ZBP1 in porcine PTCs. We subjected a PPV inoculum to an inactivating UV dose of 900 J/m^2^, and the results showed that the relative viral titers of UV-inactivated PPV decreased by 5.13 logs compared to unirradiated PPV (Figure 6A). We infected porcine PTCs with untreated PPV or UV-inactivated PPV at a MOI of 1 for 0, 24, 48, and 72 h in the absence of Ac-DEVD-CHO. The UV-inactivated PPV resulted in the release of significantly less LDH than unirradiated PPV in porcine PTCs at all the time points (Figure 6B). Western blot results revealed that the expression of ZBP1, p-RIPK3, and p-MLKL in PTCs cells infected with UV-inactivated PPV did not increase with prolonged infection time, unlike in cells infected with the non-irradiated virus. The expression of ZBP1, p-RIPK3, and p-MLKL in PTCs infected with UV-inactivated PPV was significantly lower than that in cells infected with untreated PPV (Figure 6C). Subsequently, we transfected porcine PTCs with equal amounts of PPV genome plasmids and initiation codon-mutated PPV genome plasmids, Western blot results demonstrated that the expression levels of ZBP1, p-RIPK3, and p-MLKL in the group transfected with the initiation codon-mutated PPV increased with prolonged infection time, exhibiting a trend consistent with the PPV-transfected group (Figure 6D). These results suggest that PPV DNA mediates the necroptosis process in porcine PTCs induced by PPV infection.

## Discussion

PPV, the smallest and structurally simplest single-stranded linear DNA virus among animal viruses, is known to induce reproductive disorders in sows [13]. The dysregulation of proliferating trophoblast cells is closely associated with the occurrence and development of various reproductive disorders [14, 15]. Porcine trophoblast cells (PTCs) play a crucial role in forming the placental barrier and are the primary target cells for PPV, undergoing antiviral cell death to clear PPV and prevent vertical transmission. Our previous research demonstrated that early-stage PPV infection triggered apoptosis and autophagy in PTCs as part of the antiviral defense mechanism [1]. However, the occurrence of non-apoptotic forms of cell death in the later stages of infection has recently captured more attention. Through TEM and LDH assays, we confirmed for the first time that necroptosis occurred in PTCs during the later stages of PPV infection. We also observed an upregulation of ZBP1 expression during PPV infection; thus, a reduction of ZBP1 expression might significantly reduce PPV-induced necroptosis. PPV infection upregulated the expression of p-RIPK3 and p-MLKL, but had no impact on Caspase-8 expression. Additionally, confocal microscopy revealed a translocation of MLKL from the cytoplasm to the cell membrane at 72 h of PPV infection. These results are consistent with the hypothesis that prolonged PPV infection induces ZBP1-associated necroptosis in PTCs.

Several studies have shown that viral infection may lead to multiple modes of programmed cell death in a cellular pool, and different modes of programmed cell death may result in different pathologies [16]. In our previous research, we found that an infection with PPV at a multiplicity of infection (MOI) of 1 induced approximately 25% apoptosis in PTCs at 24 h post-infection, while another approximately 7% of cells underwent non-apoptotic cell death related to autophagy [1], and we are curious about what type is this non-apoptotic cell death. Necroptosis and apoptosis are the two crucial antiviral cell death modes in organisms [17], and the ZBP1/RIPK3/MLKL signaling pathway mediates necroptosis [18, 19]. As an innate intracellular nucleic acid sensor, ZBP1 contains an RIP homotypic interacting motif (RHIM), through which it interacts with RIPK1 and RIPK3 proteins that also contain RHIM domains [6]. Following the infection of cells by bacteria or viruses, ZBP1 recognizes heterologous nucleic acids, activates RIPK3, and forms a ZBP1-RIPK3 complex. RIPK3 then phosphorylates and oligomerizes MLKL, and the resulting MLKL oligomers translocate in part to the nucleus assisted by HSP90 or PITPα, while the rest is transferred to the cytoplasmic membrane. MLKL oligomers in the cytoplasmic membrane mediate the formation of cation channels, allowing the influx of extracellular Mg^2+^, K^+^, and Na^+^ ions [19]. MLKL oligomers also mediate the exposure of membrane phospholipids to the extracellular milieux, releasing death-inducing signals [19]. As a part of this process, the opening of the membrane ion channel protein TRPM7 permits the influx of Ca^2+^. MLKL oligomers also initiate endoplasmic reticulum stress that generates ROS and releases Ca^2+^. High levels of cytoplasmic Ca^2+^ lead to lysosomal dysfunction, more ROS production, and cytoplasmic membrane rupture. Elevated levels of ROS stimulate the opening of mPTP, alter mitochondrial membrane potential, and lead to the release of cytochrome c. Furthermore, PGAM5 on mitochondria binds to the necrosome, and this complex recruits and activates drp1 or CypD, which mediate mitochondrial fission, mPTP opening, and necroptosis with Ca^2+^ influx and plasma membrane damage. In additional, it has also been found that ZBP1 mediates RIPK3-dependent induction of apoptotic and necroptotic cell death pathways during Influenza virus (IAV) infection [8]. RIPK3 is essential for necroptosis but also governs whether a cell activates Caspase-8 and dies by apoptosis [20]. The possible mechanism is that RIPK3 interacts with another RHIM-containing protein, RIPK1, which recruits Caspase-8 via Fas-associated protein with death domains (FADD) to assemble a Casp8-FADD-cFLIP complex. Then Caspase-8 homodimerization and self-cleavage drive cell-extrinsic apoptosis [5, 21]. When Caspase-8 activity is compromised, RIPK1 and RIPK3 undergo RHIM-dependent oligomerization that promotes necroptosis [21].

Caspase-8 mediates apoptosis [22, 23], and caspase inhibition is a prerequisite for the initiation of necroptosis in most cell types. Several studies have confirmed that necroptosis can only serve as an alternative antiviral cell death mode when Caspase-8 activity is disrupted [24–26]. ZBP1 is a major inducer of necroptosis in response to infection by influenza A virus, herpesviruses, and poxviruses (such as vaccinia virus) [27, 28], and functions in both nuclear and cytoplasmic compartments to mediate necroptosis induced by virus infection [18]. In addition, studies have demonstrated that influenza A virus infection can involve both Caspase-8-dependent apoptosis and ZBP1/RIPK3/MLKL signaling pathway-mediated necroptosis [29]. However, few cells exhibit features of both forms of cell death activation simultaneously. Usually only one type of cell death, apoptosis or necroptosis, occurs [30]. For flaviviruses such as Zika virus and West Nile virus, ZBP1 serves as the primary nucleic acid sensor, inducing cell death through non-dependent antiviral effects [31]. More and more studies investigate that virus infection could lead to multiple modes of programmed cell death in one cell pool, and the distinct programmed cell death modes may lead to different pathologies [16]. Normally, apoptosis is a non-inflammatory programmed cell death, while necroptosis and pyroptosis are inflammatory programmed cell deaths [32–34]. Histopathological changes indicate that PPV infection induces massive infiltration of inflammatory cells in the endometrium and lamina propria of the uterus of gilts [3], suggesting that PPV infection leads to inflammatory programmed cell death of target cells.

Very few research studies on the specific death pathway and the mechanism of its induction in PTCs infected by PPV have been done, however. In this study, we showed that PPV infection upregulated ZBP1 expression, and ZBP1 interference or knockout reduced the degree of necroptosis in the PTCs. Additionally, PPV infection enhanced the formation of p-RIPK3 and p-MLKL, but had no effect on the activation of Caspase-8. The knockout of RIPK3 did not affect the upregulation of ZBP1 induced by PPV infection but impeded the activation of MLKL. Depletion of MLKL had no impact on the PPV-induced upregulation of ZBP1 or the phosphorylation of RIPK3. Based on these findings, we conclude that PPV infection induces necroptosis in PTCs through the ZBP1/RIPK3/MLKL signaling pathway, independent of Caspase-8.

The accumulation of unstable intracellular Z-form DNA activates ZBP1, which interacts with receptor-interacting protein kinase 3 (RIPK3), to trigger an RIPK3-dependent necroptotic signaling cascade [35]. Vaccinia virus (VACV) competes with the Z-form RNA generated during VACV infection through the Zα domain at the N-terminus of its E3 protein, thus inhibiting ZBP1-mediated ZBP1/RIPK3/MLKL-dependent necrotic apoptosis [18]. A small molecule (3a-3l) based on quinoline induced DNA damage in cancer cells, and the resulting Z-form DNA inhibited the necrotic apoptosis mediated by apoptosis-inducing ZBP1 [12]. In this study, we demonstrated that UV irradiation of the virus with an inactivating dose significantly reduced PPV-induced necroptosis; initiation codon mutations did not affect PPV-induced necroptosis through the ZBP1/RIPK3/MLKL pathway, however. These results indicate that during the infection process, ZBP1 recognizes and binds to PPV DNA, thereby initiating the ZBP1/RIPK3/MLKL signaling pathway and inducing necrotic apoptosis in PTCs.

In conclusion, this study discovered and confirmed that PPV infection induced ZBP1-dependent necroptosis, providing novel insights into the antiviral mechanisms of porcine placental trophoblast cells and suggesting potential new therapeutic strategies for preventing reproductive disorders in pigs.

## Supplementary Information

**Additional file 1. PPV infection induces necroptosis of PTCs with the absence of apoptosis inhibition**. PTCs were infected with 1 MOI PPV for 0, 24, 48, and 72 h, and the LDH release levels were measured. *P < 0.05, **P < 0.01 versus cells infected by PPV for 0 h.

**Additional file 2. PPV infection in PTCs without inhibition of apoptosis can activate necroptosis**. PTCs were infection with 1 MOI PPV for 0, 24, 48, and 72 h, and the expression levels of ZBP1, p-RIPK3, RIPK3, p-MLKL, MLKL, and Caspase-8 were determined by Western blot and calculated. **P < 0.01 versus cells infected by PPV for 0 h.

**Additional file 3. siZBP1-759 significantly reduced the levels of ZBP1 mRNA and protein expression in porcine PTCs.** (A) Level of ZBP1 mRNA in the three transfected cell lines. (B) Protein expression of ZBP1 in the three transfected cell lines.

**Additional file 4. Viability of the three cell lines, 352PTCs**^***zbp1+/+***^, 368PTCs^***zbp1+/+***^, 403PTCs^***zbp1-/-***^, was similar to WT cells. The CCK-8 assay was used to measure viability of the ZBP1-knockout cell lines.

**Additional file 5. The RIPK3 knockout PTCs were generated using RIPK3-418 successfully**. (A) Sequencing of the RIPK3 locus amplified from the 481PTCs^*ripk3-/-*^ and WT cells. Black dashes indicate the bases deleted from the gene. The results showed that multiple gene deletions occurred in the RIPK3 genome of 481PTCs^*ripk3-/-*^ cells. (B) The CCK-8 assay was used to measure the viability of 481PTCs^*ripk3-/-*^.

**Additional file 6. The MLKL knockout PTCs were generated using MLKL-126 successfully. **(A) Sequencing of the MLKL locus amplified from the 126PTCs^*mlkl-/-*^ and WT cells. Black dashes indicate the bases deleted from the gene. The results showed that multiple gene deletions occurred in the RIPK3 gene of 126PTCs^*mlkl-/-*^ cells. (B) The CCK-8 assay was used to measure the viability of 126PTCs^*mlkl-/-*^.

**Additional file 7. Guide RNA sequences used in this study.**

**Additional file 8. Primer sequences for gene knockout identification used in this study**.
