# Supplementary figures and images for: Porcine parvovirus infection induces necroptosis of porcine placental trophoblast cells via a ZBP1-mediated pathway
Source: Vet Res. 2024 Nov 29;55:156. doi: 10.1186/s13567-024-01410-x (PMC11605877; doi:10.1186/s13567-024-01410-x)

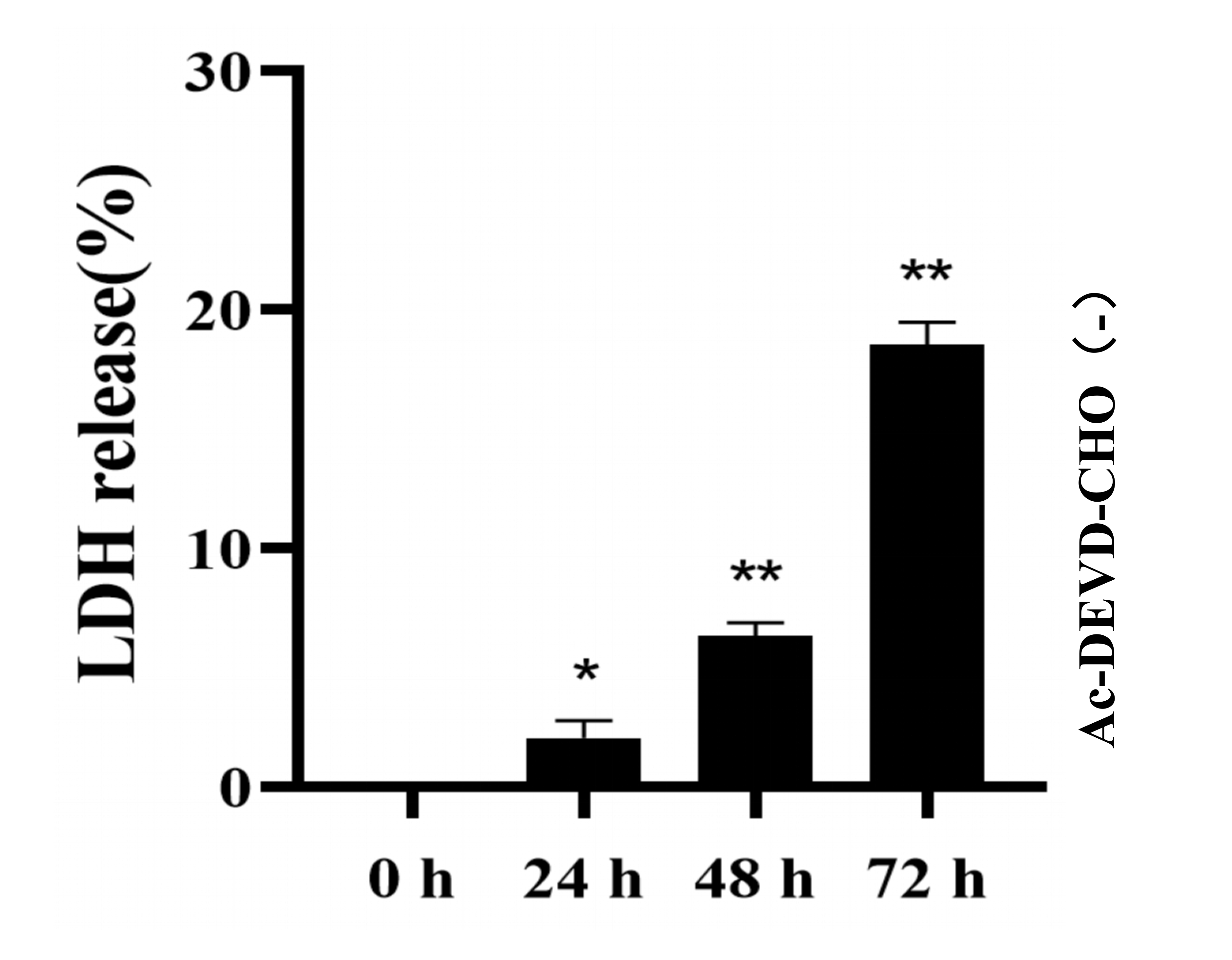

Supplement: Supplementary file 1 — Additional file 1. PPV infection induces necroptosis of PTCs with the absence of apoptosis inhibition. PTCs were infected with 1 MOI PPV for 0, 24, 48, and 72 h, and the LDH release levels were measured. *P < 0.05, **P < 0.01 versus cells infected by PPV for 0 h. [file 13567_2024_1410_MOESM1_ESM.tif]

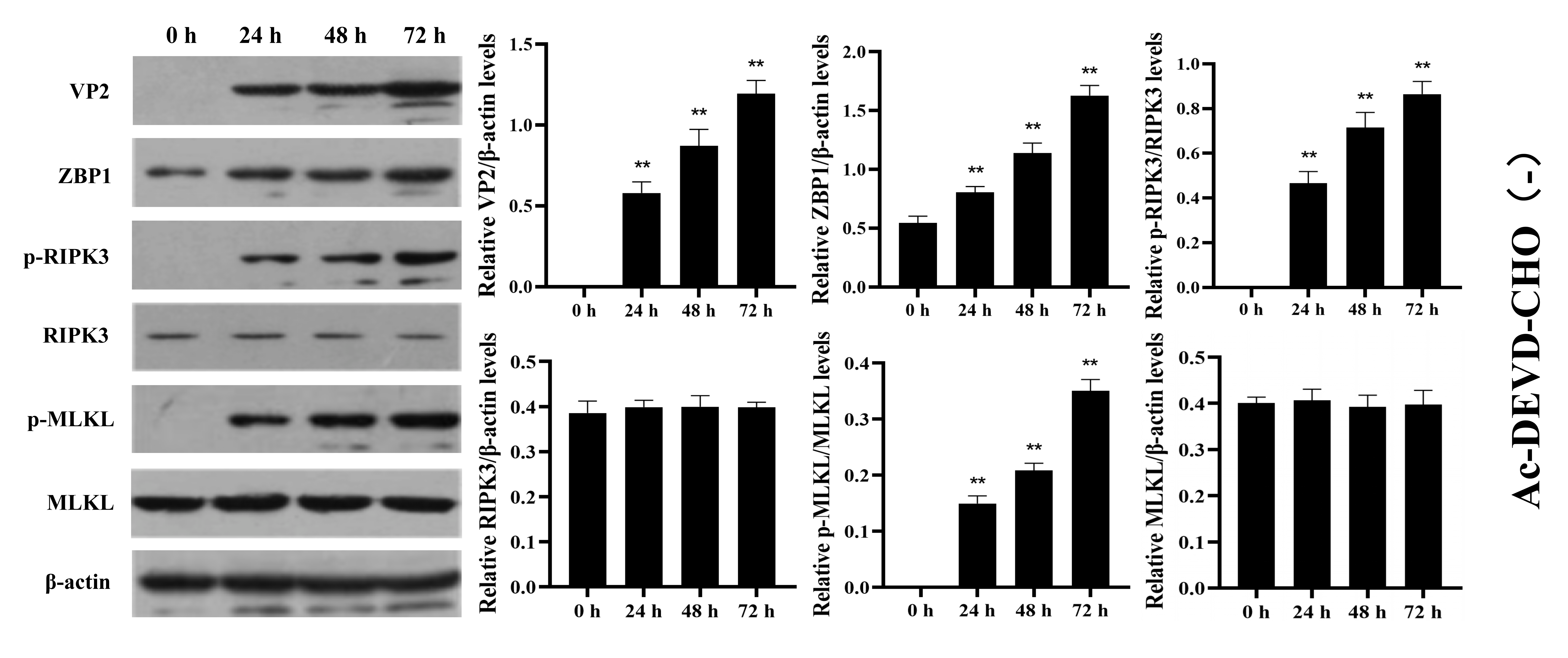

Supplement: Supplementary file 2 — Additional file 2. PPV infection in PTCs without inhibition of apoptosis can activate necroptosis. PTCs were infection with 1 MOI PPV for 0, 24, 48, and 72 h, and the expression levels of ZBP1, p-RIPK3, RIPK3, p-MLKL, MLKL, and Caspase-8 were determined by Western blot and calculated. **P < 0.01 versus cells infected by PPV for 0 h. [file 13567_2024_1410_MOESM2_ESM.tif]

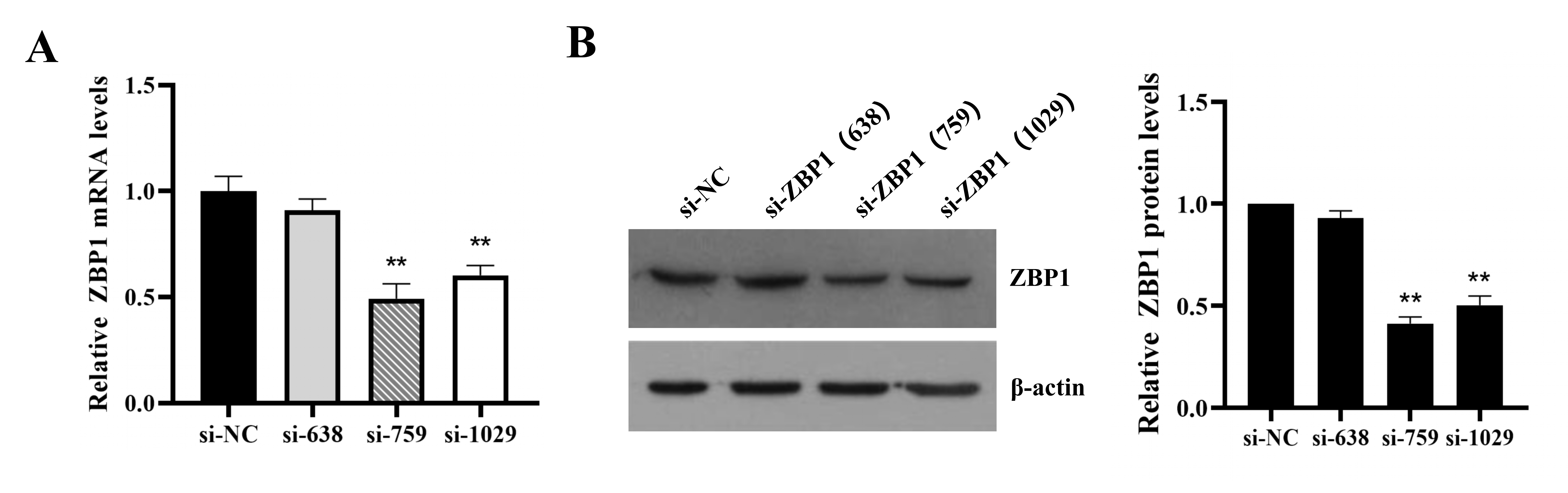

Supplement: Supplementary file 3 — Additional file 3. siZBP1-759 significantly reduced the levels of ZBP1 mRNA and protein expression in porcine PTCs. (A) Level of ZBP1 mRNA in the three transfected cell lines. (B) Protein expression of ZBP1 in the three transfected cell lines. [file 13567_2024_1410_MOESM3_ESM.tif]

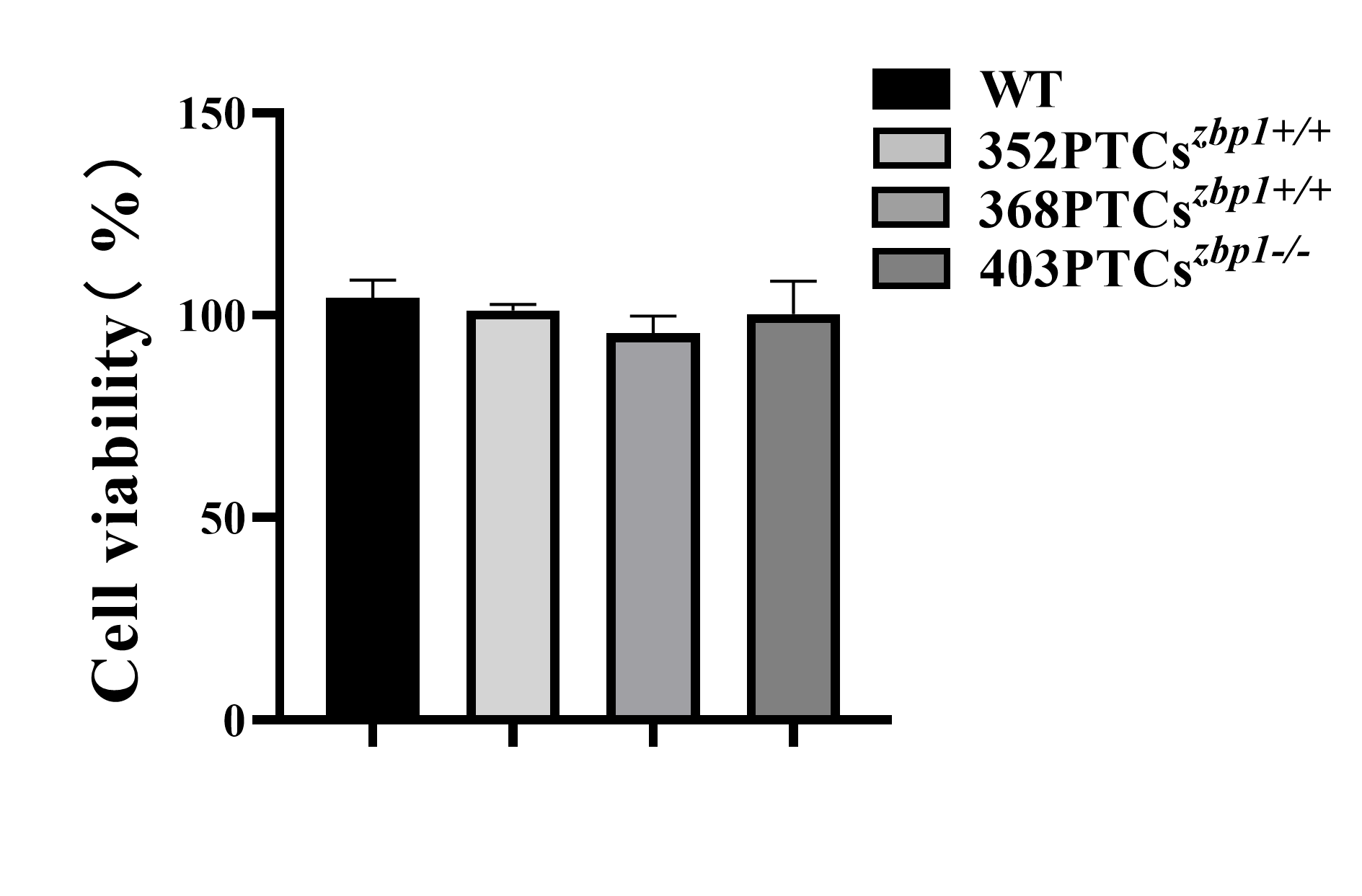

Supplement: Supplementary file 4 — Additional file 4. Viability of the three cell lines, 352PTCszbp1+/+, 368PTCszbp1+/+, 403PTCszbp1-/-, was similar to WT cells. The CCK-8 assay was used to measure viability of the ZBP1-knockout cell lines. [file 13567_2024_1410_MOESM4_ESM.tif]

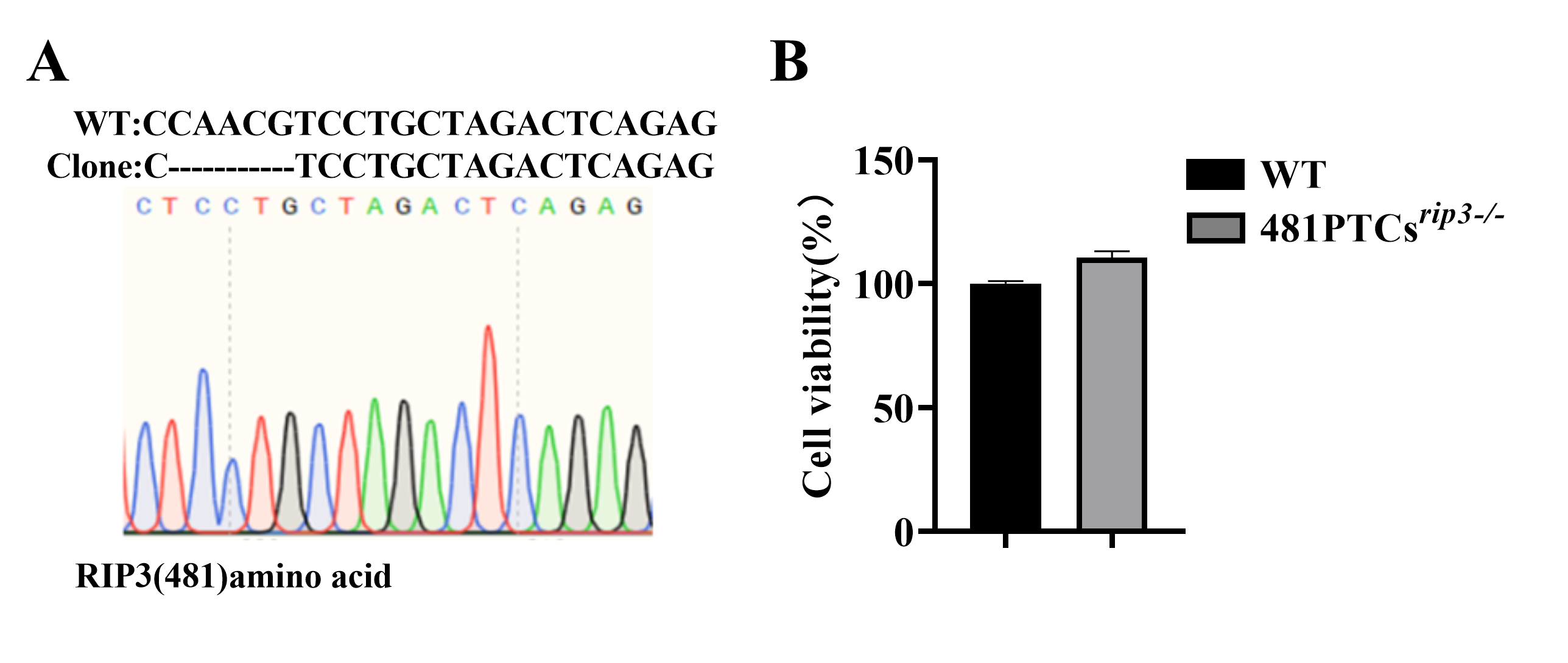

Supplement: Supplementary file 5 — Additional file 5. The RIPK3 knockout PTCs were generated using RIPK3-418 successfully. (A) Sequencing of the RIPK3 locus amplified from the 481PTCsripk3-/- and WT cells. Black dashes indicate the bases deleted from the gene. The results showed that multiple gene deletions occurred in the RIPK3 genome of 481PTCsripk3-/- cells. (B) The CCK-8 assay was used to measure the viability of 481PTCsripk3-/-. [file 13567_2024_1410_MOESM5_ESM.tif]

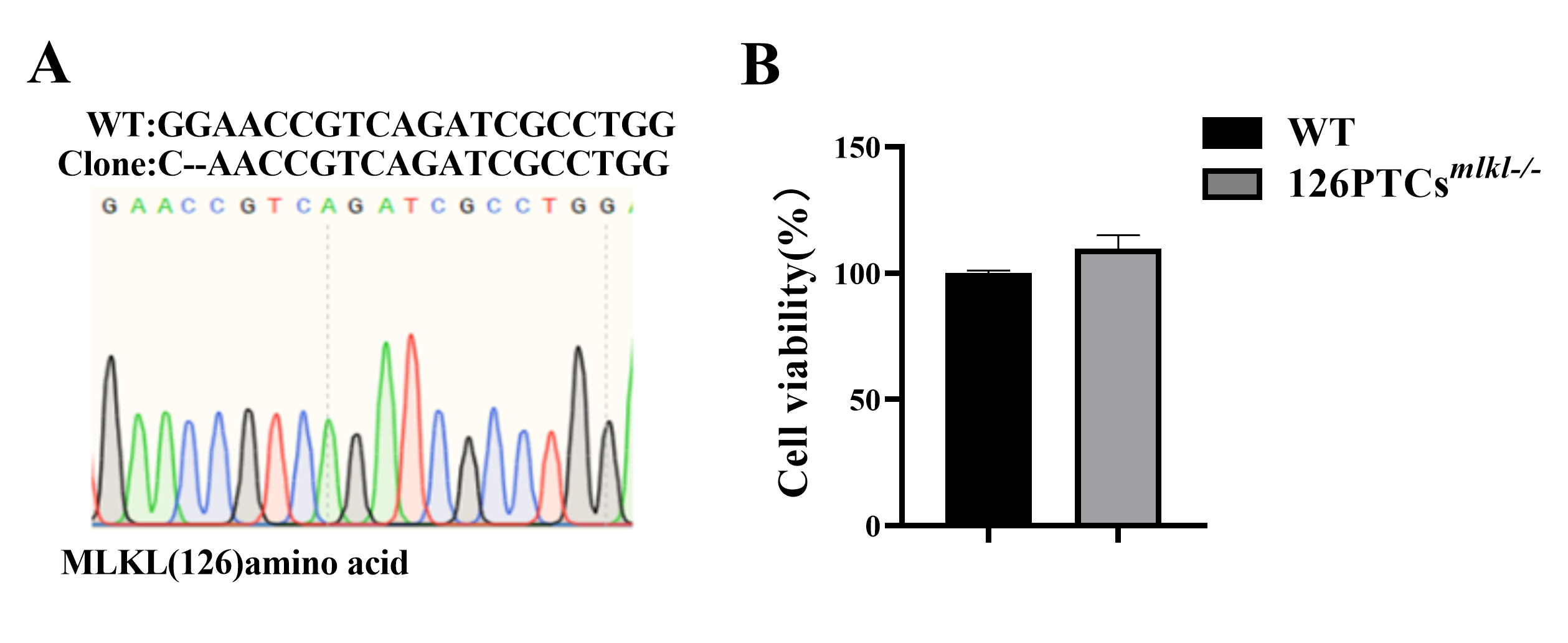

Supplement: Supplementary file 6 — Additional file 6. The MLKL knockout PTCs were generated using MLKL-126 successfully. (A) Sequencing of the MLKL locus amplified from the 126PTCsmlkl-/- and WT cells. Black dashes indicate the bases deleted from the gene. The results showed that multiple gene deletions occurred in the RIPK3 gene of 126PTCsmlkl-/- cells. (B) The CCK-8 assay was used to measure the viability of 126PTCsmlkl-/-. [file 13567_2024_1410_MOESM6_ESM.tif]
